# Supplementary material for: Reducing the Formation of Toxic Byproducts During the Photochemical Release of Epinephrine
Source: J Xenobiot. 2025 Jan 8;15(1):8. doi: 10.3390/jox15010008 (PMC11755455; doi:10.3390/jox15010008)
Supplement: Supplementary file 1 [file jox-15-00008-s001.zip › jox-3352177-supplementary.pdf]

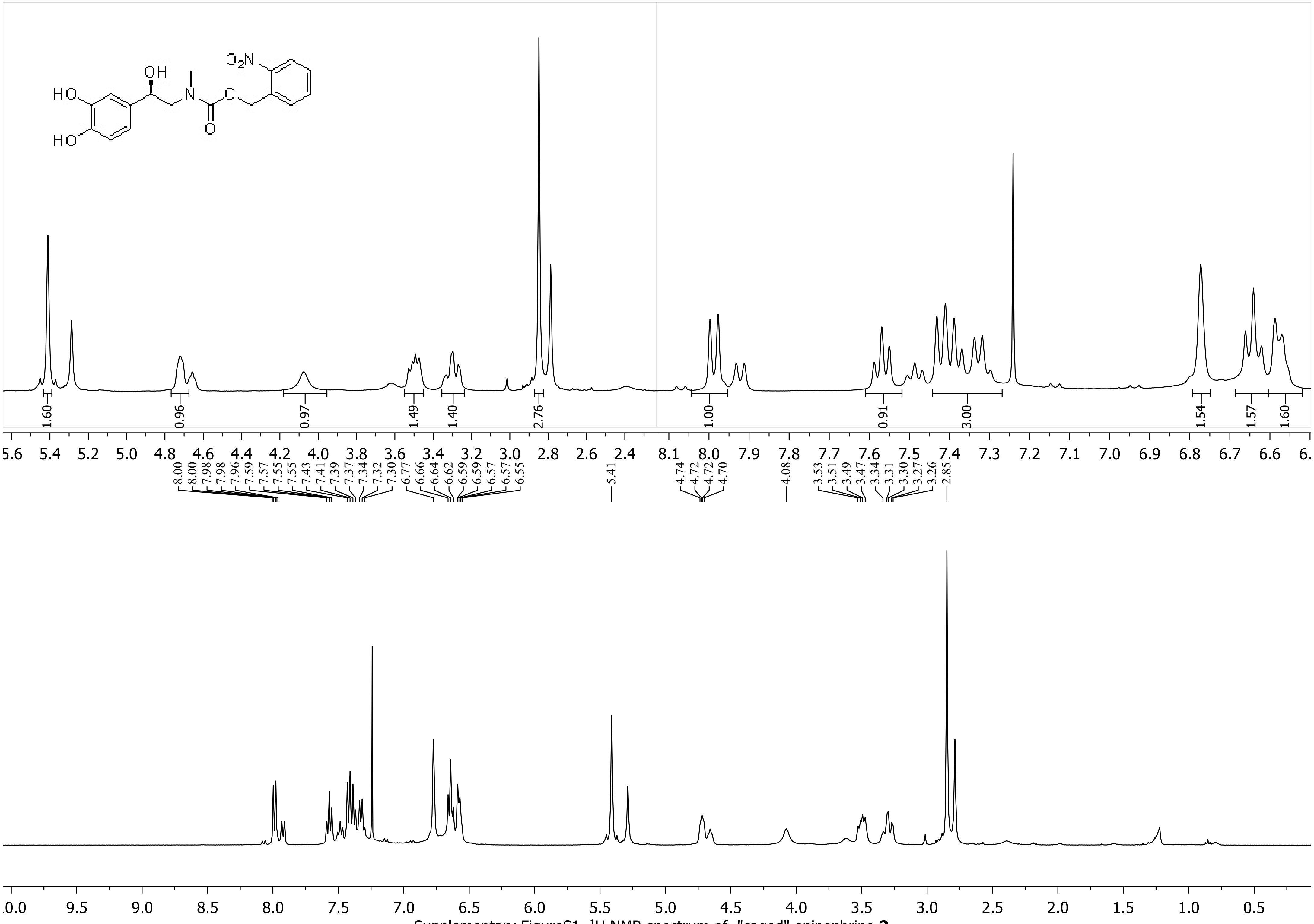

Supplementary FigureS1. ¹H NMR spectrum of "caged" epinephrine **2**

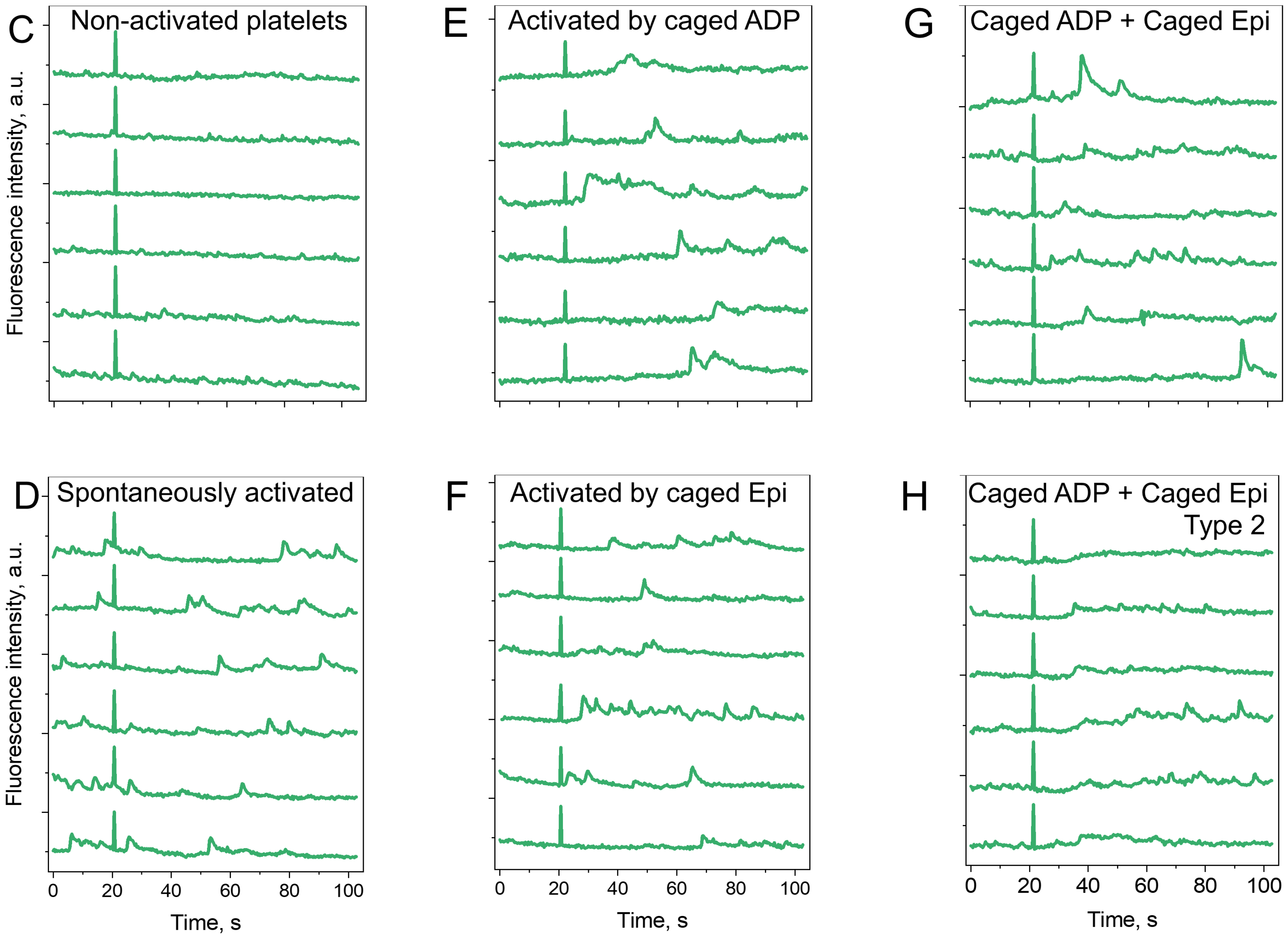

Supplementary FigureS2. Examples of single-platelet calcium dynamics. Sharp pulse at 20s. indicates the photoactivation flash.

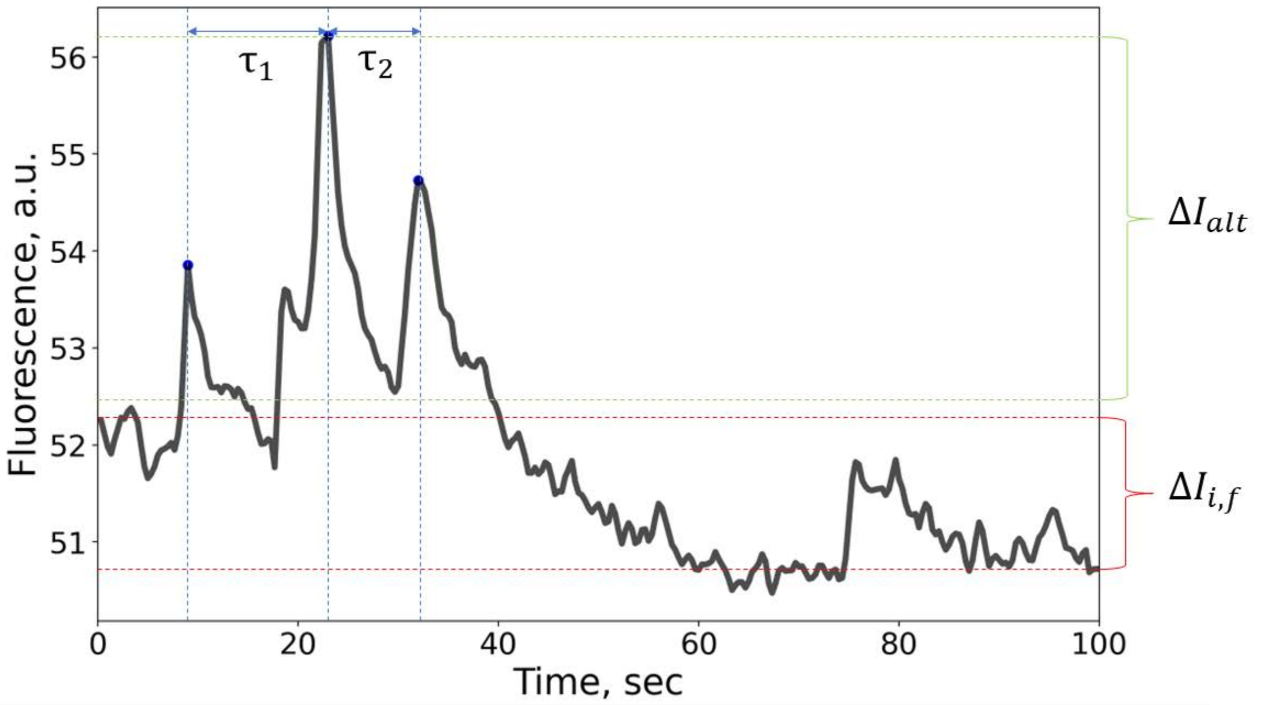

Supplementary Figure S3. Illustration of the calcium dynamics, peak detection algorithm and statistical analysis.

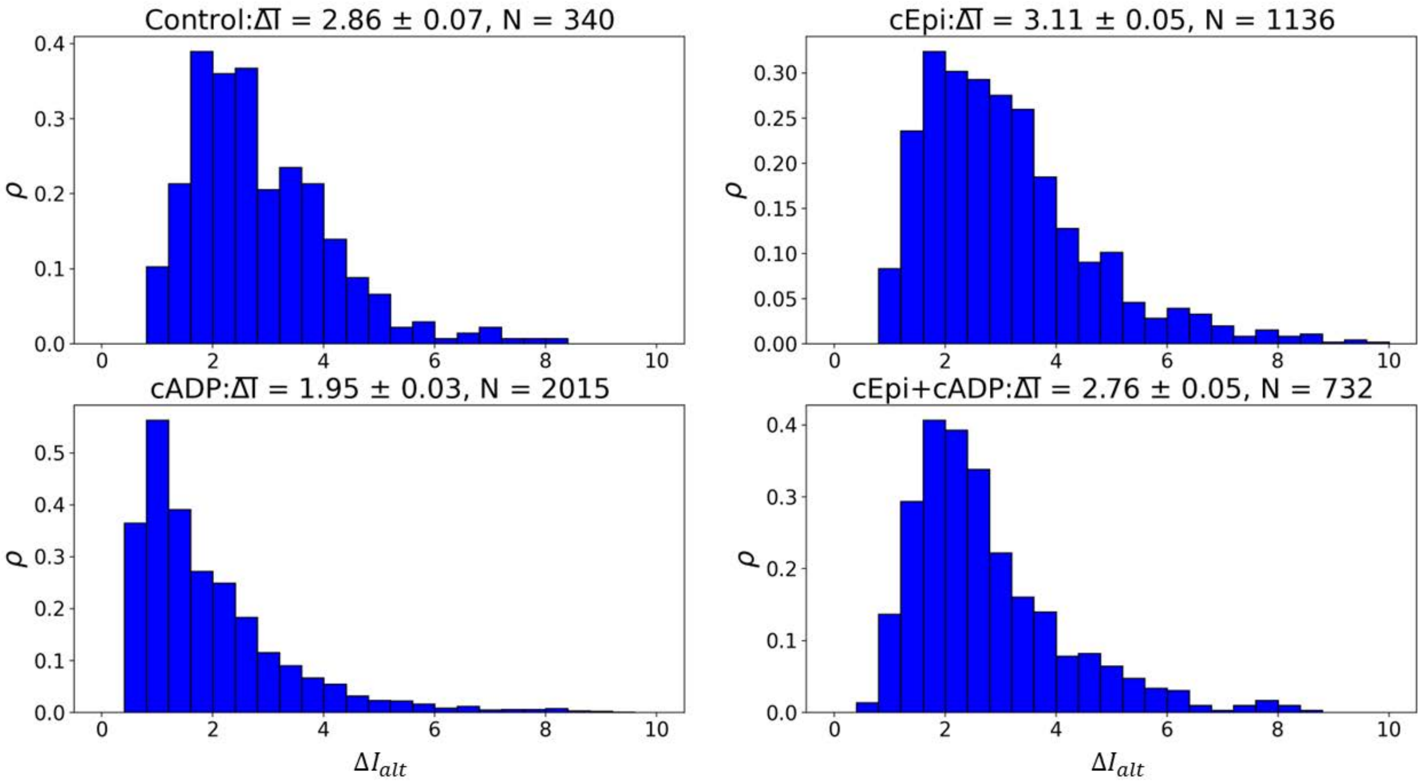

Supplementery Figure S4. Distribution of platelets over calcium spike intensity (Maximal Intensity *minus* Mean Intensity).

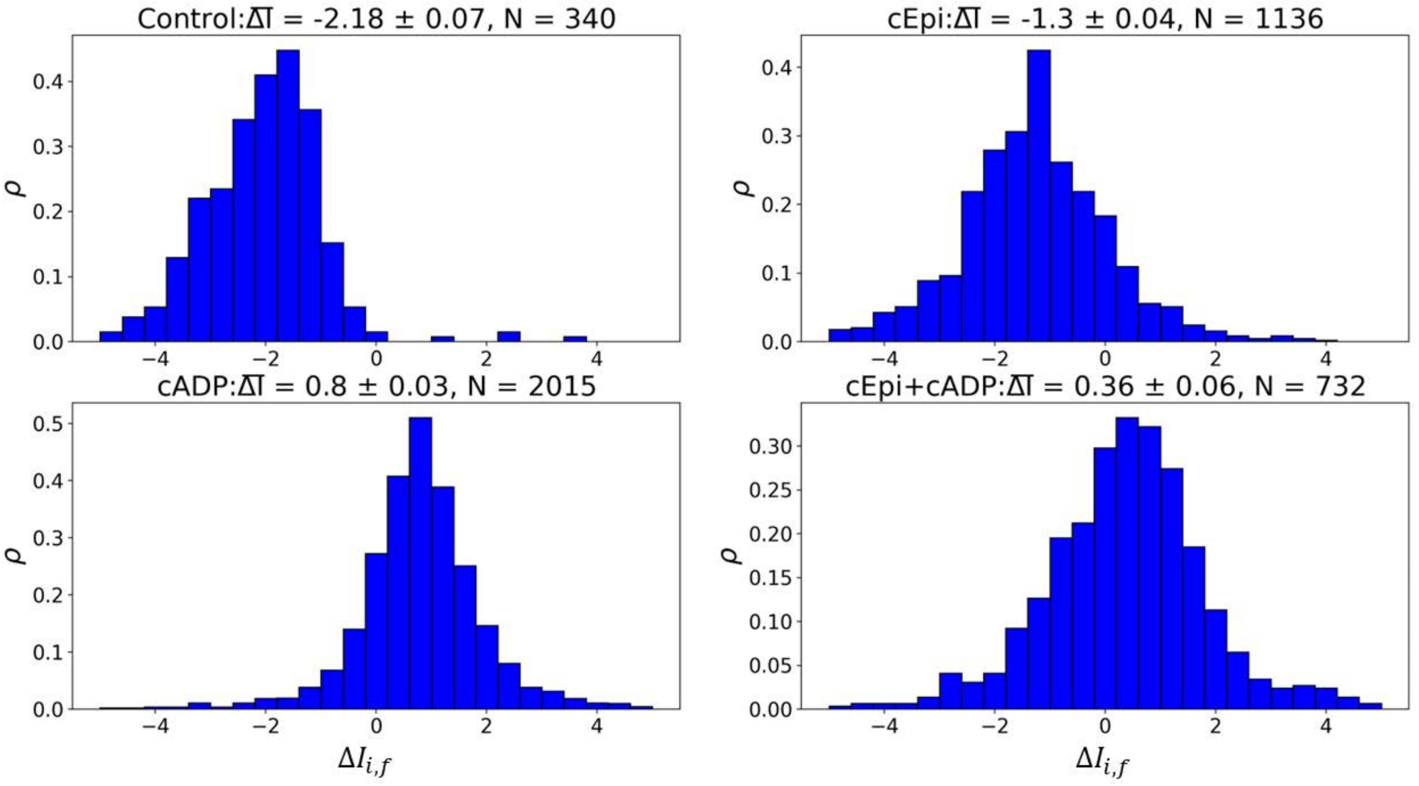

Supplementary Figure S5. Distribution of platelets over mean fluorescence intensity change (Final Intensity *minus* Initial Intensity)

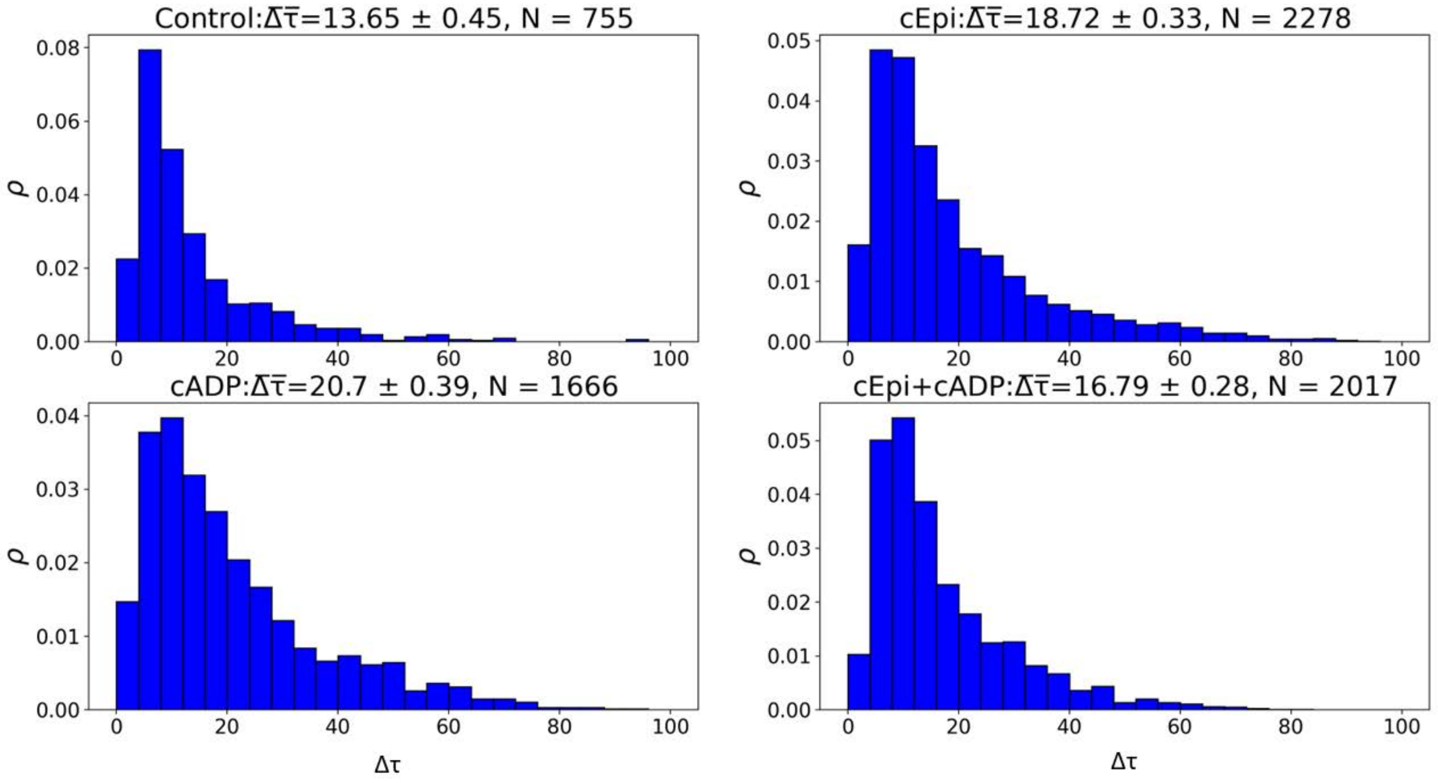

Supplementary Figure S6. Distribution of peak-to-peak intervals for platelets in all samples.
